# Preventive measures and perceived challenges in delivering oral health care for elderly patients: a survey of dental hygienists in Norway

**DOI:** 10.2340/aos.v84.42581

**Published:** 2025-01-06

**Authors:** Anne Breivik, Simen E. Kopperud, Qalbi Khan, Aida Mulic, Linda Stein

**Affiliations:** a Faculty of Social and Health Sciences, Inland Norway University of Applied Science, Elverum, Norway; b Nordic Institute of Dental Material (NIOM), Oslo, Norway; c Faculty of Dentistry, University of Oslo, Oslo, Norway; d The Artic University of Norway, Tromsø, Norway

**Keywords:** Preventive measures, oral health, root caries, elderly patients, dental hygienists

## Abstract

**Objective:**

This study aimed to gain knowledge of general oral health preventive measures with a specific focus on root caries preventive measures for patients ≥65 years old, performed by Norwegian dental hygienists in public and private dental health services. A secondary aim was to investigate differences and challenges in prevention practices.

**Materials and methods:**

An electronic survey was conducted among the sample in 2022. A total of 365 dental hygienists were included in the analyses. Chi-square tests were used to analyze differences between private and public dental hygienists regarding preventive measures and perceived challenges.

**Results:**

The most frequently reported general oral health preventive measures were oral hygiene instruction, professional tooth cleaning and scaling. Oral hygiene instruction and application of fluoride varnish were the most performed root caries preventive measure, and reduced manual dexterity in patients was the most perceived challenge. Public dental hygienists perceived challenges to a greater extent than private dental hygienists, particularly related to reduced mobility and ergonomic difficulties in patients.

**Conclusion:**

This study confirms dental hygienists’ important role in oral health promotion and showed that Norwegian dental hygienists performed a wide range of preventive measures for patients ≥65 years old. However, a number of challenges were identified in the preventive work.

## Introduction

The world’s population is aging, and the share of people aged 65 years or above is projected to accelerate in the coming decades [1]. Similar trends are expected in Norway, and during the next decade, the population will consist of more older people aged 65+ than children and young people [2]. The demographic shift will have implications for both health and oral health [3]. Oral health is an integral part of healthy aging, as it is an intrinsic constituent of general health and well-being [4, 5]. Numerous associations between general health and oral health have been identified and indicate that systemic diseases prevalent in older people such as diabetes, cardiovascular disease, and dementia can increase the risk of oral diseases [3, 6]. Risk factors for oral diseases accumulate throughout life, and older people will continuously need preventive dental services [7]. To meet the oral health needs of the aging population, WHO’s Global Oral Health Status Report (2022) highlights that oral health care services and dental professionals should provide appropriate and preventive dental services [5].

Globally, the presence of natural teeth among older adults has resulted in more dental caries, particularly root caries [8, 9]. The prevalence has been high in most Western studies on older adults [9–13], and root caries is predicted to become one of the main challenges in future dentistry [11]. Traditional restorative treatment of root caries is often challenging due to difficulties with moisture control or reduced anatomical accessibility. In addition, many older people who develop root caries have limited mobility, which makes conventional restorative treatment impossible. Consequently, there is a great need for effective and preventive root caries strategies [14].

Providing oral health care to older people is often challenging due to psychical and cognitive decline, multimorbidity, polypharmacy, and reduced mobility [15]. Internationally, a range of barriers have been reported by oral health professionals in delivering oral health care to older people, such as refusal of care, mobility challenges, or time constraints [15–18]. As preventive strategies and measures are effective at all ages, oral health professionals should provide a good standard of preventive care across the age range [19].

Dental hygienists play a vital role in oral health promotion and oral disease prevention [20]. In the Nordic countries, dental hygienists have contributed considerably to oral health care, particularly among children and adolescents [21]. Dental hygienists constitute approximately 9% of all employed in the Norwegian dental services, and the ratio of dental hygienists is 2.0 per 10.000 inhabitants nationally [22]. According to Statistics Norway, 605 dental hygienists are affiliated in public sector and 448 in the private sector [23]. Preventive dental services in Norway are authorized in the legislation, and the county municipality is obligated to promote oral health and organize preventive measures for the entire population [24]. However, to date, limited knowledge of preventive practices carried out to the elderly population in Norway exists [25]. In addition, to the best of our knowledge, studies identifying dental hygienists’ challenges in providing preventive dental services to older people are scarce.

Thus, the present study aimed to gain knowledge of general oral health preventive measures with a specific focus on root caries preventive measures for patients ≥65 years old, performed by Norwegian dental hygienists in public and private dental health services. A secondary aim was to investigate differences and challenges in prevention practices between public and private dental hygienists.

## Materials and methods

### Study design and population

**Figure 1:**
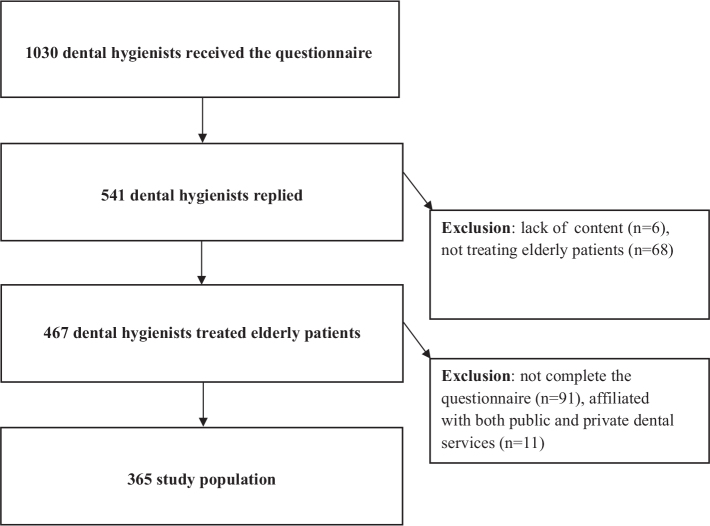
Flowchart of the study population.

This cross-sectional study was conducted on a sample of Norwegian dental hygienists registered as members of The Norwegian Dental Hygienist Federation in autumn 2022. An online survey was sent to all registered dental hygienists working in public and private sectors in Norway (*n* = 1,044), and geographically all counties were represented among the participants (data not shown). However, 14 were rejected because of invalid email addresses; therefore, 1,030 received the questionnaire. The survey used the online questionnaire software ‘Nettskjema’ developed by the University of Oslo. The second part contained a series of questions related to clinical treatment of elderly patients. Up to three automatic reminders were sent to participants not responding to the questionnaire from September to December 2022. Of the 1,030 dental hygienists that received the questionnaire, 541 participants replied to the questionnaire, leaving a response rate of 52.5%. However, as shown in Figure 1, 176 participants were further excluded due to different reasons (e.g., affiliated with both public and private dental services, making it difficult to know whether the measures were performed in public or private practise). Thus, the included sample consisted of responses from a total of 365 dental hygienists, resulting in an inclusion rate of 35.4% (Figure 1). For characteristics of the participants, see Table 1.

**Table 1:** Background characteristics of the study population (*n* = 365).

Variables	Public dental service (*n* = 234) % (*n*)	Private dental service (*n* = 131) % (*n*)	Total (*n* = 365) % (n)
**Gender**			
Female	96.2 (225)	97.7 (128)	96.7 (353)
Male	3.8 (9)	2.3 (3)	3.3 (12)
**Age groups**			
≤30 years	17.1 (40)	13.7 (18)	15.9 (58)
30–40 years	27.8 (65)	42.0 (55)	32.9 (120)
41–50 years	25.2 (59)	23.7 (31)	24.7 (90)
51–60 years	19.7 (46)	13.7 (18)	17.5 (64)
>60 years	10.2 (24)	6.9 (9)	9.0 (33)
**Graduation period**			
1970–1989	14.1 (33)	7.6 (10)	11.8 (43)
1990–1999	17.5 (41)	22.1 (29)	19.2 (70)
2000–2009	26.1 (61)	23.7 (31)	25.2 (92)
2010–2022	42.3 (99)	46.6 (61)	43.8 (160)
Working district*			
Urban	47.0 (110)	70.2 (92)	55.3 (202)
Rural	53.0 (124)	29.8 (39)	44.7 (163)

* Urban = ≥20.000 inhabitants, rural = ≤19.999 inhabitants.

### Questionnaire and measures

The questionnaire design was based on a review of relevant literature and international practice guidelines [17, 18, 26–30]. The questionnaire was two-fold; the first part collected data on the respondents, such as age, gender, occupation and whether they treated patients ≥65 years old. The second part contained questions about preventive measures and perceived challenges. Most questions were mandatory, except for a few open-ended questions, such as ‘Do you perform other root caries preventive measures than mentioned above’, and ‘Do you experience other challenges than mentioned above’.

The questionnaire was piloted twice, both manually and digitally by 20 dental hygienists with extensive experience in public and private dental services, before it was administrated to the participants.

### Statistical analyses

Descriptive analyses were performed to describe the sample and the participant’s characteristics. Chi-square tests were used to evaluate the differences between the dental hygienists working in private and public dental services regarding general oral health preventive measures, specific root caries preventive measures, and perceived challenges. The significance level was set to *p* < 0.05. All analyses were performed using IBM SPSS Statistics version 27.

### Ethical considerations

The study was approved by the Norwegian Centre for Research Data (NSD) before data collection (Reference number: 903101). The survey was distributed by a mailing list from the Norwegian Dental Hygienist Federation, which was treated as strictly confidential. Only the project manager had access to the e-mail addresses while the data collection was in progress. The email addresses were deleted and replaced with an identification number when the data collection had ended, and before analysing data.

## Results

The distribution of general oral health preventive measures performed by dental hygienists in public and private dental services is shown in Table 2. The results showed that public dental hygienists mapped dietary habits (*p* < 0.001), performed Mucosal Plaque Score (*p* < 0.001), and applied of Fluoride Varnish^®^ (*p* < 0.001) significantly more often than private dental hygienists. Similarly, private dental hygienists conducted saliva testing (*p* < 0.001), professional tooth cleaning (*p* = 0.003), and periodontal probing depths (*p* < 0.001) significantly more often than public dental hygienists.

**Table 2:** How often do you perform the following preventive measures in patients ≥65 years old?.

Preventive measures	Public dental service (*n* = 234)	Private dental service (*n* = 131)	Total (*n* = 365)	*P*
	% (*n*)	% (*n*)	% (*n*)	
**Mapping dietary habits**				**<0.001**
Always or often Occasionally or never	32.1 (75) 67.9 (159)	12.2 (16) 87.8 (115)	24.9 (91) 75.1 (274)	
**Mapping oral hygiene habits**				0.596
Always or often	88.9 (208)	87.0 (114)	88.2 (322)	
Occasionally or never	11.9 (26)	13.0 (17)	11.8 (43)	
**Oral hygiene instruction**				0.568
Always or often	94.9 (222)	96.2 (126)	95.3 (348)	
Occasionally or never	5.1 (12)	3.8 (5)	4.7 (17)	
**Mucosal-Plaque Score (MPS/BSI)**				**<0.001**
Always or often	82.1 (192)	36.6 (48)	65.8 (240)	
Occasionally or never	17.9 (42)	63.4 (83)	34.2 (125)	
**Plaque and bleeding index**				0.105
Always or often	53.8 (126)	62.6 (82)	57.0 (208)	
Occasionally or never	46.2 (108)	37.4 (49)	43.0 (157)	
**Saliva test if indicated hyposalivation**				**<0.001**
Always or often	16.2 (38)	32.1 (48)	21.9 (80)	
Occasionally or never	83.8 (196)	67.9 (89)	78.1 (285)	
**Application of fluoride varnish**				**<0.001**
Always or often	83.3 (195)	51.1 (67)	71.7 (262)	
Occasionally or never	16.7 (39)	48.9 (64)	28.2 (103)	
**Fluoride gel in trays**				0.264
Always or often	0.4 (1)	1.5 (2)	0.8 (3)	
Occasionally or never	99.6 (233)	98.5 (129)	99.2 (362)	
**Professional tooth cleaning**				**0.003**
Always or often	91.9 (215)	99.2 (130)	94.5 (345)	
Occasionally or never	8.1 (19)	0.8 (1)	5.5 (20)	
**Periodontal probing depths**				**<0.001**
Always or often	59.0 (138)	96.2 (126)	72.3 (264)	
Occasionally or never	41.0 (96)	3.8 (5)	27.7 (101)	
**Scaling**				0.184
Always or often	92.7 (217)	96.2 (126)	94.0 (343)	
Occasionally or never	7.3 (17)	6.0 (5)	16.7 (22)	

The distribution of specific root caries preventive measures performed by public and private dental hygienists is illustrated in Table 3. The results show that private dental hygienists performed plaque removal (*p* < 0.001), prevention of gingival recession (*p* < 0.001) and helped patients in quit smoking (*p* < 0.001) significantly more often than public dental hygienists, while public dental hygienists performed application of Fluoride Varnish^®^ (*p* = 0.015) significantly more often than private dental hygienists. Regarding the open- ended answer option; ‘do you perform other root caries preventive measures than mentioned above’, the majority of those who replied, reported the use of Duraphat toothpaste^®^ (data not shown).

**Table 3:** Which root caries preventive measures do you perform in patients ≥65 years old?.

Preventive measures	Public dental service % (*n*)	Private dental service % (*n*)	Total % (*n*)	*P*
Dietary instruction	59.4 (139)	49.6 (65)	55.9 (204)	0.071
Oral hygiene instruction	97.9 (229)	99.2 (130)	98.4 (359)	0.332
Application of fluoride varnish	89.3 (209)	80.2 (105)	86.0 (314)	**0.015**
Plaque removal	57.3 (134)	87.8 (115)	68.2 (249)	**<0.001**
Prevention of gingival recession	56.8 (133)	87.8 (115)	67.9 (248)	**<0.001**
Helping patients quit smoking	12.8 (30)	28.2 (37)	18.4 (70)	**<0.001**
Fluoride gel in trays	1.3 (3)	0	0.8 (3)	0.193
Chlorhexidine mouthwash	11.5 (27)	13.7 (18)	12.3 (45)	0.539
Chlorhexidine gel in trays	0.4 (1)	0.8 (1)	0.5 (2)	0.677

Participants only answered if the specific root caries preventive measure was performed.

Table 4 presents the participants perceived challenges in the preventive work. Overall, the results indicated that public dental hygienists experienced challenges to a greater extent than private dental hygienists. Significant differences were found in almost all given challenges (Table 4). Regarding the open-ended answer option; ‘do you experience other challenges than mentioned above’, 18 public dental hygienists replied. The majority reported oral health care in nursing homes to be challenging, for example, low priority among health personnel, lack of routines and time constraints (data not shown). Among the private dental hygienists, 6 participants replied, and described difficulties such as multiple diseases in patients and low willingness to change oral health habits in patients to be challenging (data not shown).

**Table 4:** To which extent do you experience the following challenges in the preventive work in patients ≥65 years old?

Challenges	Public dental service (*n* = 234) % (n)	Private dental service (*n* = 131) % (n)	Total (*n* = 365) % (n)	*P*
**Time constraints**				
To a great extent	55.6 (130)	19.1 (25)	42.5 (155)	**<0.001**
To less extent	44.4 (104)	80.9 (106)	57.5 (210)	
**Lack of adequate dental equipment**				
To a great extent	32.9 (77)	6.1 (8)	23.3 (85)	**<0.001**
To less extent	67.1 (157)	93.9 (123)	76.7 (280)	
**Lack of dental professionals**				
To a great extent	50.0 (117)	9.2 (12)	35.3 (129)	**<0.001**
To less extent	50.0 (117)	90.8 (119)	64.7 (236)	
**Mobility challenges patient**				
To a great extent	82.1 (192)	23.7 (31)	61.1 (223)	**<0.001**
To less extent	17.9 (42)	76.3 (100)	38.9 (142)	
**Ergonomic challenges patient**				
To a great extent	74.8 (174)	22.1 (29)	55.6 (203)	**<0.001**
To less extent	25.6 (60)	77.9 (102)	44.4 (162)	
**Reduced dexterity patient**				
To a great extent	91.0 (213)	72.5 (95)	84.4 (308)	**<0.001**
To less extent	9.0 (21)	27.5 (36)	15.6 (57)	
**Financial constraints patient**				
To a great extent	21.4 (50)	45.0 (59)	29.9 (109)	**<0.001**
To less extent	73.6 (184)	55.9 (72)	70.1 (256)	
**Patient having pain/discomfort**				
To a great extent	32.5 (76)	22.1 (29)	28.8 (105)	**0.036**
To less extent	67.5 (158)	77.9 (102)	71.2 (260)	
**Odontophobia**				
To a great extent	12.0 (28)	19.1 (25)	14.5 (53)	0.064
To less extent	88.0 (206)	80.9 (106)	85.5 (312)	
**Low health literacy patient**				
To a great extent	67.5 (158)	41.2 (54)	58.1 (212)	**<0.001**
To less extent	32.5 (76)	58.8 (77)	41.9 (153)	
**Low health literacy informal caregivers**				
To a great extent	38.0 (89)	16.8 (22)	30.4 (111)	**<0.001**
To less extent	62.0 (145)	83.2 (109)	69.6 (254)	
**Low health literacy health personnel**				
To a great extent	59.0 (138)	34.4 (45)	50.1 (183)	**<0.001**
To less extent	41.0 (96)	65.6 (86)	49.9 (182)	
**Negative oral health attitudes health personnel**				
To a great extent	57.7 (135)	20.6 (27)	44.4 (162)	**<0.001**
To less extent	42.3 (99)	79.4 (104)	55.6 (203)	
**Negative oral health attitudes informal caregivers**				
To a great extent	13.2 (31)	11.5 (15)	12.6 (46)	0.620
To less extent	86.8 (203)	58.5 (116)	87.4 (319)	
**Negative oral health attitudes patient**				
To a great extent	32.9 (77)	21.4 (28)	28.8 (105)	**0.020**
To less extent	67.1 (157)	78.6 (103)	71.2 (260)	
**Limitations in own level of knowledge/competence**				
To a great extent	19.2 (45)	26.0 (34)	21.6 (79)	0.135
To less extent	80.7 (189)	74.0 (97)	78.4 (286)	
**Lack of national guidelines**				
To a great extent	29.1 (68)	32.1 (42)	30.1 (110)	0.549
To less extent	70.9 (166)	67.9 (89)	69.9 (255)	
**Own language barriers**				
To a great extent	3.0 (7)	3.1 (4)	3.0 (11)	0.973
To less extent	97.0 (227)	96.9 (127)	97.0 (354)	
**Language barriers patient**				
To a great extent	12.8 (30)	6.1 (8)	10.4 (38)	**0.044**
To less extent	87.2 (204)	93.9 (123)	89.6 (327)	
**Lack of consent competence** (e.g. patients having dementia)				
To a great extent	51.3 (120)	12.2 (16)	37.3 (136)	**<0.001**
To less extent	48.7 (114)	87.8 (115)	62.7 (229)	
**Patients not attending their appointments**				
To a great extent	26.9 (63)	10.7 (14)	21.1 (77)	**<0.001**
To less extent	73.1 (171)	89.3 (117)	78.9 (288)	

## Discussion

This study aimed to gain knowledge of general oral health preventive measures and specific root caries preventive measures for patients ≥65 years old, performed by Norwegian dental hygienists in public and private dental health services. A secondary aim was to investigate differences and challenges in prevention practices between public and private dental hygienists. Our results demonstrated that Norwegian dental hygienists performed a wide range of preventive measures, showing that preventive care as a mean for oral health improvement is widely emphasised among older patients. The most frequently reported general oral preventive measures were oral hygiene instruction, professional tooth cleaning, scaling, and mapping of oral hygiene habits. This is in accordance with results from a Scottish survey by Turner et al., which showed that particularly oral hygiene instruction and scaling were central measures given to older patients by public and private dental hygienists [18]. Oral hygiene instruction and scaling are also found to be among the most frequently reported preventive measures given by dental hygienists internationally [26, 31].

In contrast, our results showed that mapping of dietary habits, saliva tests, and fluoride gel in trays were performed less frequently. The most surprising finding in this regard was that only one-fourth of the dental hygienists reported that they always or often mapped dietary habits, although national guidelines for oral health care to adults recommend that adults regularly should be given dietary guidance in accordance with national dietary advices [32]. A similar outcome was found in the study by Turner et al., which reported that dietary guidance had low priority among public and private dental hygienists. This was also the case in a recent Norwegian survey investigating oral health care for older adults in home health care services, which found that 15% of dental hygienists reported never giving dietary advice, and less than half stated that they often gave dietary advice [33]. As dietary intake often is compromised in older people [34], this is an worrying finding indicating a need to strengthen the focus on dietary habits among older people in the dental health services.

The most performed root caries preventive measures by both groups were oral hygiene instruction and the application of fluoride varnish (Duraphat^®^), utilised by nearly all dental hygienists. In addition, both groups reported Duraphat high-fluoride toothpaste (5,000 ppmF) as a central ‘other’ root caries preventive measure. These findings largely correspond to recommended non-invasive methods identified to reduce the development of root caries lesions in elderly people [35–40]. Due to these findings, the results indicate that the utilised root caries preventive strategies could have an advantageous effect in the prevention of root caries among elderly patients in the Norwegian dental health services. Another issue to consider alongside the clinical effects of the preventive measures is cost-effectiveness. Preventive measures might reduce the need for even more costly restorative treatment, particularly as invasive treatment might fail repeatedly [14]. A cost-effectiveness perspective of root caries prevention should be relevant for policymakers, both on the clinical level and on the healthcare service level.

Several differences regarding preventive measures were identified between the two groups. The most prominent differences were that public dental hygienists significantly more often reported to perform application of Duraphat^®^ varnish and mucosal-plaque score (MPS) compared to private dental hygienists, while private dental hygienists significantly more often performed saliva tests and periodontal probing depths. According to root caries preventive measures, significantly more private dental hygienists performed plaque removal, prevention of gingival recession, and gave advice on smoke cessation compared to public dental hygienists. Similar differences are previously confirmed in other Nordic studies investigating dental hygienists working profiles, demonstrating that public and private dental hygienists perform different working tasks [26, 41, 42]. In general, the identified differences can be explained by the two-fold oral health care system in Norway. The public dental service is state-funded and provides oral health care to patients prioritised in The Dental Health Services Act, such as children under the age of 18 and elderly people in institutional and domiciliary care [24]. The private dental service mainly treats the adult population who must pay for the dental services themselves [43]. Due to the organizational structure of the Norwegian dental service, it is not unexpected that dental hygienists spend their working time with different groups of older patients, and that variations in the distribution of working task are present. For example, as MPS is a common tool to improve oral health and determine the quality of implemented measures for older people in institutional care in Norway [44], it is not surprisingly that the public dental hygienists performed this measure significantly more often than the private dental hygienists. Likewise, it is not unexpected that private dental hygienists significantly more often performed periodontal probing depths in comparison to public dental hygienist, as periodontal therapy is a central working task in private practise. However, the different preventive strategies in public and private sectors are considered as a benefit rather than a disadvantage, as the various strategies can complement each other in the different phases of the elderly’s life course.

The participants perceived a range of challenges that hampered the provision of oral health care. Reduced manual dexterity in patients was the most experienced challenge by both groups. This is an important finding, because as the population ages, many will face challenges with mechanical plaque removal due to reduced manual dexterity associated with health conditions such as severe arthritis, Parkinson disease or dementia [3, 45–47]. Consequently, many people with these diagnoses will be in need of oral health care [48]. Therefore, both oral health professionals and caregivers responsible for oral health in older people must be aware of manual dexterity as a predictor for poor oral health [49, 50].

Dental hygienists working in the public dental services perceived more challenges compared to dental hygienists in the private dental services. The most prominent finding was seen regarding reduced mobility and ergonomic difficulties in patients, which was reported almost four times more often by public dental hygienists compared to private dental hygienists. Similar challenges were experienced by dental hygienists in the study by Turner et al., which highlighted that mobility issues in older people who, for example, needed to be treated in wheelchairs often caused limitations in the dental treatment [18]. Our results also correspond to findings from the study by Uhlen-Strand et al., which found that a large proportion of public oral health professionals reported ergonomic issues as challenging in the provision of oral care to elderly patients [33]. Considering the different work responsibility of public and private dental hygienists, the results are not unexpected. Due to the public dental hygienists’ statutory role in treating disabled elderly patients in institutional or domiciliary care, it is reasonable that they faced more challenges related to these patient groups compared to the private dental hygienists.

The identified challenges still raise some concerns regarding the organizational structure of the Norwegian dental service, and one might speculate whether the dental service is prepared to cope with these challenges as the aging populations needs for dental services are increasing. This is further reinforced by future perspectives indicate that dental hygienists’ preventive strategies targeting older people will be more complex in the coming decades. Trends indicate that the professional roles of dental hygienists will expand in line with the aging population and their demand for preventive dental services. Multiple chronic medical conditions will increase the complexity of oral health care given to older people, and future dental hygienists must be prepared to work with medically compromised patients in multiple settings [27, 51, 52]. In addition, higher expectations of oral health and the awareness of the relationship between oral health and general health will all contribute to a larger need for multiskilled dental hygienists promoting preventive oral health care to the population [27]. Consequently, future dental hygienists are expected to have a broader role in multidisciplinary settings, and their scope of practise may expand to include minimally invasive restorative care or minor surgical dental care [51, 53, 54]. These perspectives are important to consider when allocating resources for future dental services.

As with any other questionnaire studies, the present study has some built-in limitations. Firstly, the anonymous design of the questionnaire does not allow for collecting information from the non-responders. Although our response rate (52.5%) should be considered decent, the inclusion rate was only 35.4% due to the exclusion criteria. Thus, the potential of non-response bias cannot be excluded, and generalizability of our findings should be made by caution. Nevertheless, the distribution of the respondents in the present study is a strength, as all counties in Norway were represented and included dental hygienists working in both rural and urban districts. Another strength is that nearly all questions in the survey were mandatory, resulting in few missing data. It has been argued that questionnaire studies may not provide accurate data because the answers are limited by the respondent’s ability to recall [55]. One limitation is response bias subjected to social desirability bias, leading to responses that are ‘politically’ correct according to guidelines or experts’ opinions. Another limitation is the nature of non-validated self-reports that may not reflect the actual behavior. Nevertheless, other studies have demonstrated high concordance between questionnaire responses and actual treatment previously [56].

In conclusion, the results showed that dental hygienists in public and private dental services performed a wide range of oral health preventive measures and root caries preventive measures for patients ≥65 years old, although differences were reported among the two groups. The findings also indicate that dental care for older people is challenging. Public dental hygienists perceived challenges to a greater extent than private dental hygienists, particularly related to reduced mobility and ergonomic difficulties in patients. It might be reasonable to call for more resources in the dental services, particularly to care for the growing population of frail elderly persons having their natural dentition. Although dental hygienists play an essential role in oral health promotion and oral disease prevention, interdisciplinary collaboration is required. Future studies should investigate how dental care could be better implemented in the general care for elderly patients.
